# Lipid profile as a novel prognostic predictor for patients with acute myeloid leukemia

**DOI:** 10.3389/fonc.2023.950732

**Published:** 2023-01-31

**Authors:** Shenrui Bai, Huizhong Wang, Ruonan Shao, Bibo Fu, Shujing Lu, Jingzi Wang, Yue Lu, Hua Wang

**Affiliations:** State Key Laboratory of Oncology in South China, Collaborative Innovation Center for Cancer Medicine, Sun Yat-sen University Cancer Center, Guangzhou, Guangdong, China

**Keywords:** acute myeloid leukemia, lipid profile, blood chemical analysis, prognostic factors, predictive modeling, dyslipdemia

## Abstract

**Purpose:**

This study investigated the relationship between serum lipid levels and clinical outcomes in acute myeloid leukemia (AML) by establishing a predictive risk classification model.

**Method:**

A total of 214 AML patients who were pathologically diagnosed and treated with standard induction chemotherapy at Sun Yat-Sen University Cancer Center were included. The patients were randomly divided into the training (n = 107) and validation (n=107) cohorts. Univariate and multivariate Cox analyses were used to assess the value of triglyceride (TG), Apolipoprotein B (Apo B), Apo Apolipoprotein A-I (Apo A-I), cholesterol (CHO), and high-density lipoprotein (HDL) as prognostic factors for AML.

**Results:**

After a series of data analyses, a five-factor model was established to divide the patients into high- and low-risk groups. Kaplan-Meier survival analysis showed that the high-risk group had a poor prognosis (*P*<0.05). The area under the curve of the novel model for five-year OS was 0.737. A nomogram was constructed to integrate the model with age and the 2017 ELN cytogenetic classification, with the merged model showing improved accuracy with an area under the curve of 0.987 for five-year OS.

**Conclusion:**

A novel model was constructed using a combination of the serum lipid profile and clinical characteristics of AML patients to enhance the predictive accuracy of clinical outcomes. The nomogram used the lipid profile which is routinely tested in clinical blood biochemistry and showed both specific prognostic and therapeutic potential.

## Introduction

Acute myeloid leukemia (AML) is known for its complicated cytogenetics and pathological heterogeneity, its strikingly high relapse tendency, and is lethal in ~50% of young adults and ∼80% of older adults (1). Owing to its poor prognosis and easy recurrence, efforts have been made to improve the standardization of chemotherapy and risk classification, such as the risk classification of the 2017 European LeukemiaNet (ELN) (2). However, the currently used prognostic risk stratification criteria for AML focus mainly on mutations seen in cytogenetic analysis and are, therefore limited in terms of accurate prognostic prediction. Improvement in accurate and individualized risk assessment is thus required.

Lipids are important components of cell membranes and have been associated with the underlying mechanisms of cancer progression, including excess proliferation and aberrant signaling. In addition, lipid metabolism plays a key role in cellular energy supply and signaling, as well as other essential aspects of tumor cell proliferation. Due to the increased metabolism and proliferation of tumor cells, dyslipidemia is typically observed in various tumor patients with a variety of tumor types (3). A series of recent studies have illustrated that the serum lipid profile (including apolipoprotein [Apo] A-1, Apo B, cholesterol [CHO], triglycerides [TG], high-density lipoprotein [HDL], and low-density lipoprotein (LDL]) is valuable in tumor prognosis prediction (4–6) and, as a result, the lipid profile has been considered as a promising therapeutic target (7–9). These studies include a report by our colleagues that demonstrated an association between lipid metabolism and the prognosis of patients with multiple myeloma (10), which made us wonder about the role of lipid metabolism in other hematological tumors. As far as we know, few studies have focused on the relationship between serum lipid levels and survival outcomes in patients with AML.

In the present study, we investigated the role of lipid and apolipoprotein profiles as prognostic indicators in AML. Not only did we retrospectively analyze the lipid characteristics of AML patients and explore their value in predicting disease prognosis when combined with current clinical indicators, but we also developed a lipid profile-based model which was found to have improved prognostic precision.

## Materials and methods

We performed a retrospective analysis of the clinical data of 273 AML patients. The inclusion criteria were patients who had been pathologically diagnosed with AML and were first treated in the Sun Yat-sen University Cancer Center (SYSUCC) between Dec 2000 and May 2021. The exclusion criteria were: a) patients with acute promyelocytic leukemia; b) patients who had previously taken or were regularly taking lipid-lowering medication, or who had a combination of chronic metabolic diseases such as diabetes mellitus, chronic renal failure, or abnormal liver function at the time of initial diagnosis; c) patients with missing lipid profile data; d) patients with incomplete follow-up data or whose survival periods were too short to analyze; e) patients with other malignant diseases. After the collection of information on treatment, a further 17 cases were excluded as they had not received the standard induction remission chemotherapy with cytarabine and DNR (Daunorubicin)/IDR(Idarubicin) (DNR 50 mg/m2, d1–3 + Ara-C 100 mg/m2, d1–7; or IDA 12 mg/m2, d1–3 + Ara-C 100 mg/m2, d1–7). Finally, 214 patients were selected for analyses (10) and were arbitrarily separated into the training (TC, n = 107) and validation cohorts (VC, n = 107).

### Clinical information and serum lipid characterization

Patient data were collected after diagnosis and before treatment. The following baseline demographics were obtained and analyzed: age, sex, white blood cell count (WBC), apolipoprotein AI (Apo A-1), apolipoprotein B (Apo B), cholesterol (CHO), triglycerides (TG), high-density lipoprotein (HDL), low-density lipoprotein (LDL), lactate dehydrogenase (LDH), body mass index (BMI), dates of diagnosis, death, or last follow-up, and cytogenetic risk classification based on the 2017 ELN criteria along with information on initial therapy. Laboratory examinations were performed on fresh blood samples obtained from patients after overnight (ON) fasting. The median duration between blood collection and treatment initiation was 14 days (3-25 days). This duration was not based on the patients’ OS.

### Follow-up and study endpoints

Following treatment, patients were monitored *via* hospital outpatient appointments or telephone conversations. Interviews were conducted once in six months for the first three years, and then annually to assess relapse or death. The last follow-up visit was dated July 31, 2021, to verify the final status of the study participants and to exclude those who could not be contacted. The primary endpoint was overall survival (OS), described as the duration of time between diagnosis and death for patients who had died or between diagnosis and the last follow-up for those that had survived.

Threshold identification for prognostic indicators

To assess possible prognostic indicators among lipid and apolipoprotein profiles, X-tile software (3.6.1)16 was used to identify the optimal OS-based threshold. The patients were then stratified into low- (LR) and high-risk (HR) sub-cohorts. Univariate and multivariate Cox regression analyses were then used to identify potential independent prognostic values associated with the lipid profile. Optimum cutoff values were determined by X-tile as follows: Age (60 years), WBC (76×10^9^/L), Apo-A1 (0.7 g/L), Apo B (0.65 g/L), CHO (2.67 mmol/L), TG (2.55 mmol/L), HDL (0.45 mmol/L), LDL (1.43 mmol/L), LDH (187.9 U/L), and BMI (19.3).

### Statistical analysis

SPSS version 26 (IBM Corporation, Armonk, NY, USA) was used for all data analyses. Continuous variables were analyzed using one-way ANOVA and categorical variables by χ^2^ or Fisher exact tests. Continuous data are presented as the mean ± standard deviation. After determining the optimal cutoff value for classifying continuous variables as categorical variables by X-tile software, univariate and multivariate Cox regression hazard models were employed to identify the independent prognostic indicators for AML patients.

Subsequently, optimal weighting coefficients for the stand-alone prognostic indicators were identified using least absolute shrinkage and selection operator (LASSO) regression analysis. OS analyses were conducted using Kaplan-Meier (KM) curves. The prognostic power of the model was determined using time-dependent receiver operating characteristic (ROC) curves and areas under the curve (AUCs). These analyses were initially conducted in the TC followed by verification in the VC. A two-tailed P-value <0.05 was set as the significance threshold. R software (version 3.6.3 for Windows, http://www.R-project.org) was used for significance estimation.

## Results

### Subject recruitment and demographics

**Figure 1:**
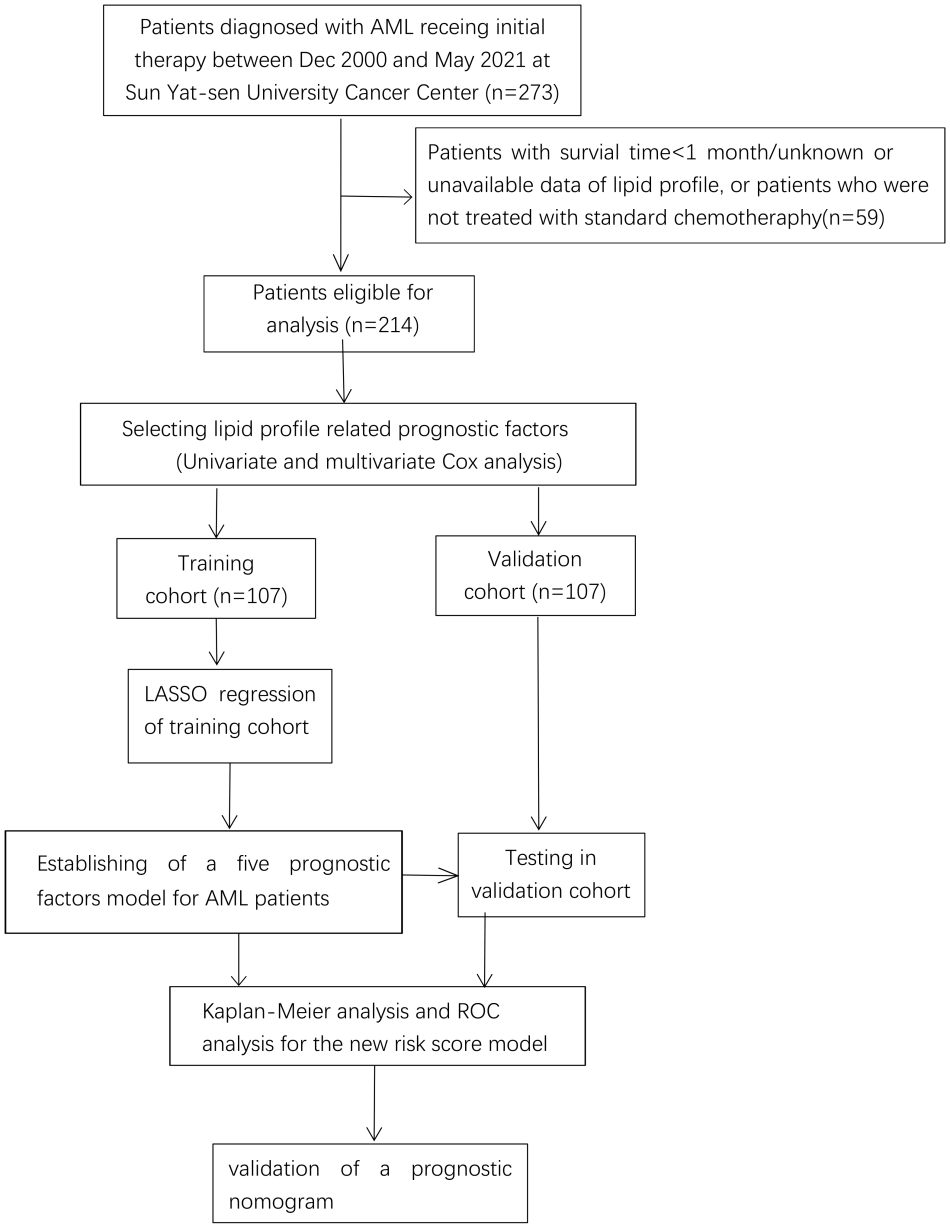
Flow chart of data collection and analysis.

A total of 214 AML patients who fulfilled the criteria for lipid and survival information were enrolled and separated into the TC (n=107) and VC (n=107) cohorts as described in the flow chart (**Figure 1**); the baseline clinical data of the patients are provided in **Supplemental Table 1**. The median age was 45 years. Ninety-one patients (42.5%) were male and 123 (57.5%) were female. According to the 2017 ELN cytogenetic risk classification, 48 patients (22.4%) were categorized as favorable, 49 patients (22.9%) as intermediate, and 63 patients (29.4%) as adverse, while the cytogenetic information on 54 patients was not available. Patients with positive mutations in the TP53 or RUNX1 genes were classified as high-risk, patients with mutations in NPM1 or CEPBA were classified as low-risk, and the remaining patients were classified as intermediate risk. The distribution of the patients’ lipid and other clinical characteristics are shown in **Table 1**. Approximately 51% of cases died before the date of the last follow-up.

**Table 1:** Univariate and multivariate COX analysis of OS in AML patients.

Variables	Hazard Ratio	Std. Err.	p	[95% Conf. Interval}	
Univariate analysis					
Gender (male vs. female)	1.188	0.187	0.356	0.824	1.712
Age (≥60 vs. <60)	3.637	0.207	**0.000**	2.426	5.452
Apo A-I (>0.7 vs. ≤0.7g/L)	0.310	0.188	**0.000**	0.214	0.448
Apo B (>0.65 vs. ≤0.65 vs.)	0.373	0.185	**0.000**	0.373	0.537
CHO (>2.67 vs.≤2.67mmol/L)	0.275	0.198	**0.000**	0.183	0.405
TG (>2.55mmol/L vs.≤2.55)	3.052	0.212	**0.000**	2.013	4.628
HDL (>0.45 vs. ≤0.45 mmol/L)	0.191	0.215	**0.000**	0.126	0.292
LDL (>1.43 vs. ≤1.43mmol/L)	0.373	0.212	**0.000**	0.246	0.565
LDH(≥250 vs. <250 U/L)	1.081	0.184	0.673	0.753	1.552
WBC (>76 vs. ≤76*10^9/L)	1.487	0.205	0.053	0.995	2.222
**Multivariate analysis**					
Age(≥60 vs. <60)	2.545	0.239	**0.000**	1.592	4.068
Apo A-I (>0.7 vs.≤0.7 g/L)	0.471	0.229	**0.001**	0.301	0.739
Apo B (>0.65 vs.≤0.65 g/L)	0.556	0.261	**0.024**	0.317	0.825
CHO (>2.67 vs.≤2.67 mmol/L)	0.525	0.371	**0.042**	0.272	0.867
TG (>2.55 vs.≤2.55 mmol/L)	2.170	0.271	**0.004**	1.275	3.694
HDL (>0.45 vs.≤0.45 mmol/L)	0.359	0.290	**0.000**	0.203	0.634
LDL (>1.43 vs.≤1.43 mmol/L)	1.605	0.330	0.152	0.840	3.065

OS, overall survival; LDH, lactate dehydrogenase; TC, total cholesterol; TG, tri-glyceride; LDL, low-density lipoprotein cholesterol; HDL, high-density lipoprotein cholesterol; Apo B, Apolipoprotein B; Apo A-I, Apo Apolipoprotein A-I. Bold fonts indicate that the p-value is statistically significant.

### Identification of independent prognostic features

The clinical characteristics were divided into categorical variables based on the optimum cut-off values, and univariate and multivariate analyses were performed to identify indices of prognostic value. Univariate analysis showed that the indices of the lipid profile, namely, Apo A-I, Apo B, CHO, TG, LDH, HDL, and LDL were significant prognostic factors (*p*<0.05) while multivariate analysis showed that Apo A-I, Apo B, TG, CHO, and HDL were independent prognostic factors **(** **Table 1**, *p*<0.05).

**Figure 2:**
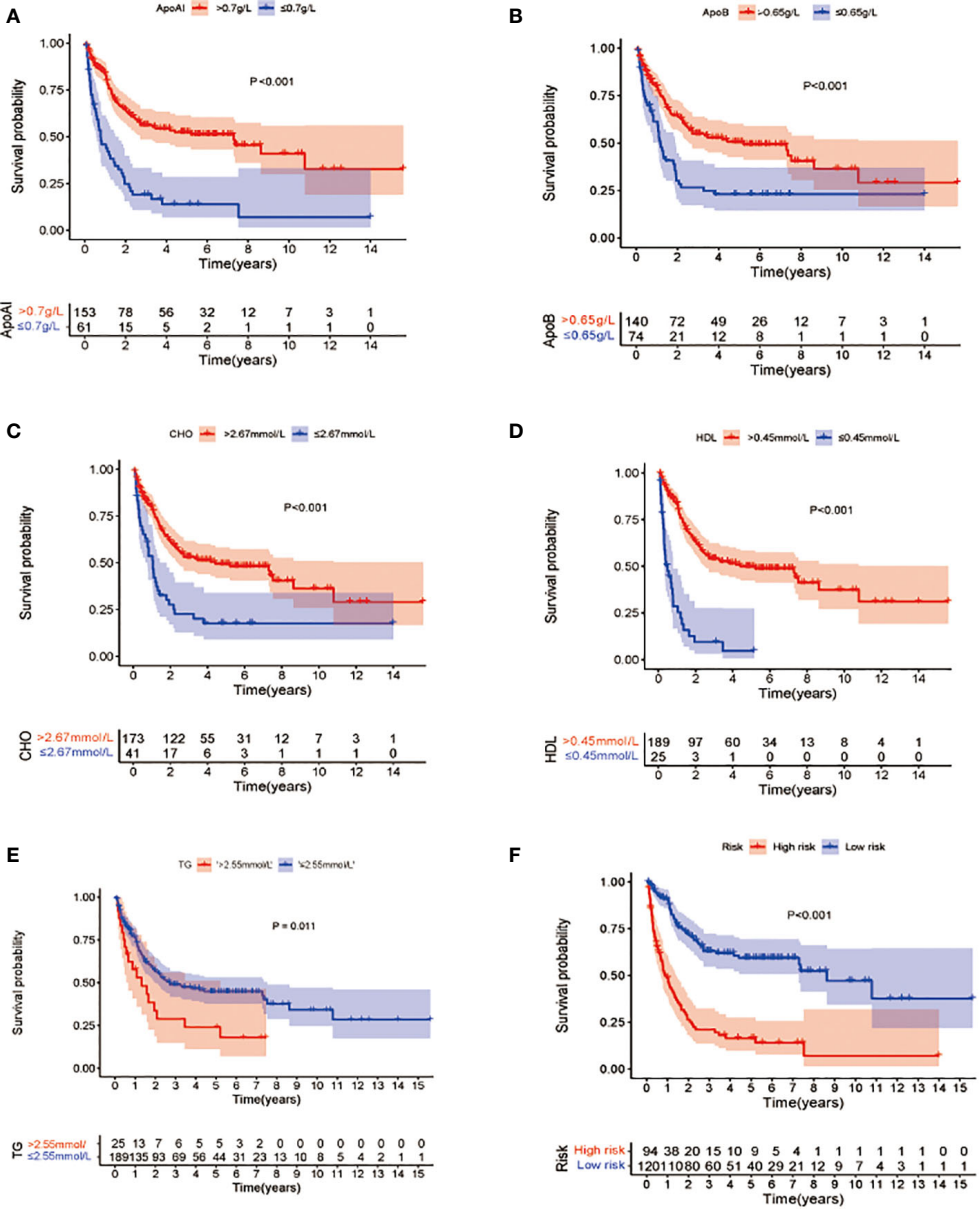
Kaplan-Meier survival analysis of overall survival between patients groups with low and high levels of lipid profile biomarkers **(A–E)**. **(A)** OS stratified by the level of Apo A-I ≤0.7 vs >0.7 g/L (p<0.001). **(B)**: OS stratified by the level of Apo B ≤0.65 vs >0.65 g/L (p<0.001). **(C)** OS stratified by the level of CHO ≤2.67 vs >2.67 mmol/L(p<0.001). **(D)** OS stratified by the level of HDL ≤0.45 vs >0.45 mmol/L (p <0.001). **(E)** OS stratified by the level of TG ≤2.55 vs >2.55 mmol/L (p<0.001). **(F)** Survival differences between high- and low-risk groups in the whole cohort.

To verify the definitions of the optimal cutoff values, KM OS analysis was used to compare the differences in OS associated with reduced and elevated lipid and apolipoprotein levels, respectively (**Figures 2A–E**). This showed a significant reduction in OS in patients with low Apo A-I, Apo B, HDL, and CHO, compared with patients with elevated levels (P<0.05) (**Figures 2A–D**), while significantly longer OS was observed in patients with lower TG levels compared with patients with high TG (*p*<0.05) (**Figure 2E**).

### Generation and verification of lipid profile risk scores (RS)

**Figure 3:**
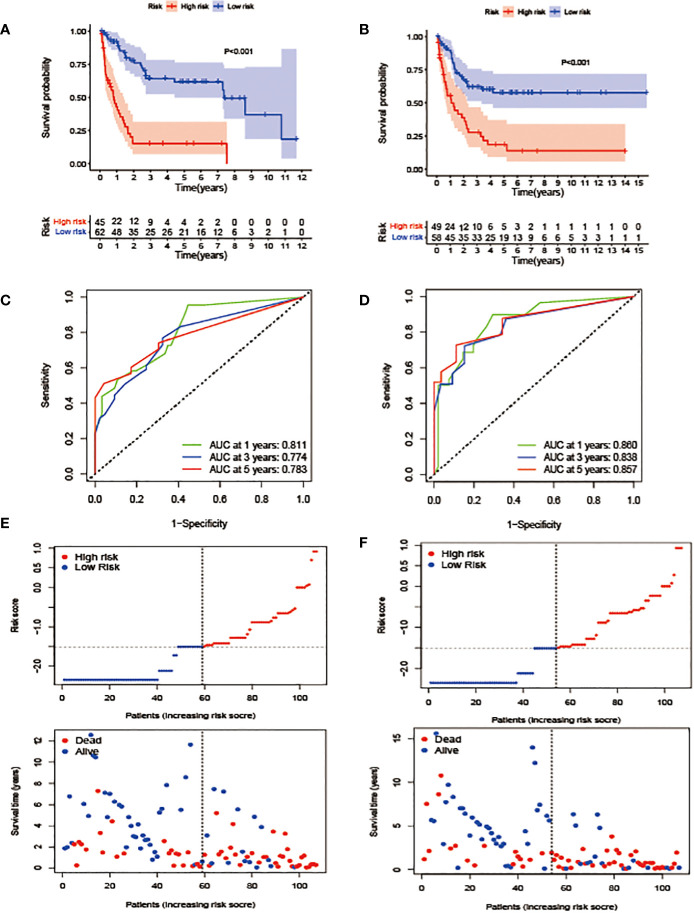
Construction and validation of the prognostic model. **(A, B)**. OS stratified by the new lipid profile risk score in the training **(A)** and validation **(B)** cohorts. **(C, D)** Areas under the curve (AUCs) of a receiver-operator characteristic (ROC) curve were compared among the one-, three-, and five-year OS of the prognostic model in the training **(C)** and validation **(D)** cohorts. Higher AUC values indicate greater prediction accuracy. **(E, F)** Risk score analysis of the signature in the high- and low-risk groups in the training and validation cohorts.

Cox analyses were first used to identify factors that were significantly associated with the prediction of prognosis. Next, LASSO regression analysis was used to generate a prognostic model using the five identified prognostic factors (Apo A-I, Apo B, TG, HDL, and CHO) in the TC. After calculation of the best weighting coefficients by the regularization parameter lambda and the 1-SE criteria (**Supplement Figure 1**), a five-factor prognostic model was selected to be included using the equation: RS= - 0.23× serum Apo A-I levels - 0.84×serum Apo B levels - 0.93×serum HDL levels -0.63× serum CHO levels + 0.93 × serum TG levels, and the RS for each AML patient was calculated as described above. The patients were then assigned to low- (LR) and high-risk (HR) groups according to the median threshold of the lipid-profile RS calculated using the TC data. After grouping, the differences between the HR and LR cohorts were analyzed by one-way ANOVA and χ^2^ tests (**Table 2**). K-M curves showed that patients with low RS values had significantly longer OS (*p*<0.01) in the entire group (**Figure 2F**), the TC (**Figure 3A**), and the VC (**Figure 3B**).

**Table 2:** The detailed characteristics of patients and correlation between clinicopathological features and risk score level in the training and validation cohorts.

Characteristics	training cohort(n=107)		P-value	Validating cohort(n=107)		P-value
	High risk, n(%)	Low risk, n(%)		High risk, n(%)	Low risk, n(%)	
**Patient**	48	59		53	54	
**age**			0.077			**0.042**
<60	36 (75.0)	52 (88.1)		39 (73.6)	48 (88.9)	
≥60	12 (25.0)	7 (11.9)		14 (26.4)	6 (11.1)	
**gender**			0.175			0.365
female	25 (52.1)	23 (39.0)		19 (35.8)	24 (44.4)	
male	23 (47.9)	36 (61.0)		34 (64.2)	30 (55.6)	
**BMI**			0.384			0.711
<19.3	15 (31.3)	14 (23.7)		14 (26.4)	16 (29.6)	
≥19.3	33 (68.8)	45 (76.3)		39 (73.6)	38 (70.4)	
**WBC**			0.408			0.314
<76	33 (68.8)	46 (78.0)		40 (75.5)	45 (83.3)	
≥76	15 (31.3)	13 (22.0)		13 (24.5)	9 (16.7)	
**Apo B**			**0.000**			**0.000**
≤0.65	32 (66.7)	11 (18.6)		37 (69.8)	10 (18.5)	
>0.65	16 (33.3)	48 (81.4)		16 (30.2)	44 (81.5)	
**Apo A1**			**0.000**			**0.001**
≤0.7	25 (52.1)	6 (10.2)		34 (64.2)	7 (13.0)	
>0.7	23 (47.9)	53 (89.8)		19 (35.8)	47 (87.0)	
**CHO**			**0.000**			**0.000**
≤2.67	15 (31.2)	2 (3.4)		27 (24.5)	8(14.8)	
>2.67	33 (68.8)	57 (96.6)		26 (75.5)	46(85.1)	
**TG**			**0.035**			**0.000**
≤2.55	31 (64.6)	59 (100.0)		34 (64.2)	54 (100.0)	
>2.55	17 (35.4)	0 (0.0)		19 (35.8)	0 (0)	
**HDL**			0.063			**0.000**
≤0.45	17 (35.4)	0 (0.0)		23 (43.4)	0(0.0)	
>0.45	31 (64.6)	59 (59.0)		30 (56.6)	54 (100.0)	
**LDH**			0.065			0.933
<187.9	25 (52.1)	41 (69.5)		30 (56.6)	31 (57.4)	56
≥187.9	23 (47.9)	18 (30.5)		23 (43.4)	23 (42.6)	
**PLR**			0.131			**0.015**
<60	29 (60.4)	27 (45.8)		35 (66.0)	23 (42.6)	
≥60	19 (39.6)	32 (54.2)		18 (34.0)	31 (57.4)	
**NLR**			0.621			0.775
<3.3	5 (10.4)	8 (13.6)		5 (84.4)	6 (11.1)	
≥3.3	43 (89.6)	51 (86.4)		48 (15.6)	48 (88.9)	
**Cytogenetic risk classification**			0.591			0.116
Favorable	8 (26.7)	16 (37.2)		6 (15.4)	18 (33.3)	
Intermediate	14 (46.7)	11 (25.6)		8 (20.5)	16 (29.6)	
Adverse	8 (26.7)	16 (37.2)		25 (64.1)	10 (18.5)	

Apo B, apolipoprotein BI; Apo A-I, apolipoprotein A-I; CHO, cholesterol; TG, triglyceride; HDL, high density lipoprotein; LDL, low density lipoprotein; LDH, lactate dehydrogenase; Cytogenetic risk classification refers to European LeukemiNet (ELN) 2017 risk classification; NLR, Neutrophil-to-Lymphocyte ratio; PLR, Platelet-to-Lymphocyte ratio. Bold fonts indicate that the p-value is statistically significant.

In terms of the evaluation of the prognostic efficiency, in the TC, the AUCs for the one-, three-, and five-year survival were 0.811, 0.774, and 0.783, respectively (**Figure 3C**), and were 0.860, 0.838, and 0.857, respectively, in the VC (**Figure 3D**), indicative of predictive significance. We also used dot plots to compare the distribution of the lipid profile RSs in patients with different OS times. The findings revealed that OS was lengthened in the low-risk group but reduced in the high-risk group (**Figures 3E, F**) in both cohorts.

### Univariate and multivariate analyses

**Figure 4:**
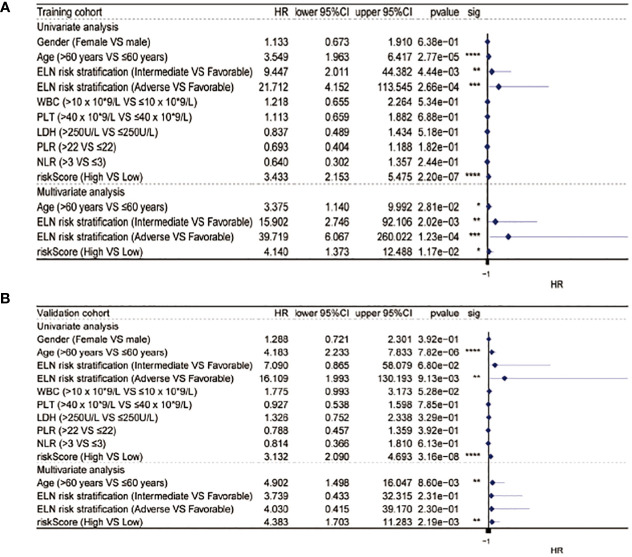
Univariate and multivariate Cox analysis in the training **(A)** and validation **(B)** cohorts. * equals p<0.05; ** equals p<0.01; *** equals p<0.001; **** equals p<0.0001.

After the measurement of the RS, we transformed the clinical features into categorical variables and repeated the univariate and multivariate analyses by incorporating the RS and other identified prognostic factors. The univariate analysis indicated that the RS independently predicted OS length and, after the removal of confounders in the multivariate analysis, the RS remained an independent predictor of OS in both cohorts (**Figure 4**).

### Comparison of the prognostic factors

**Figure 5:**
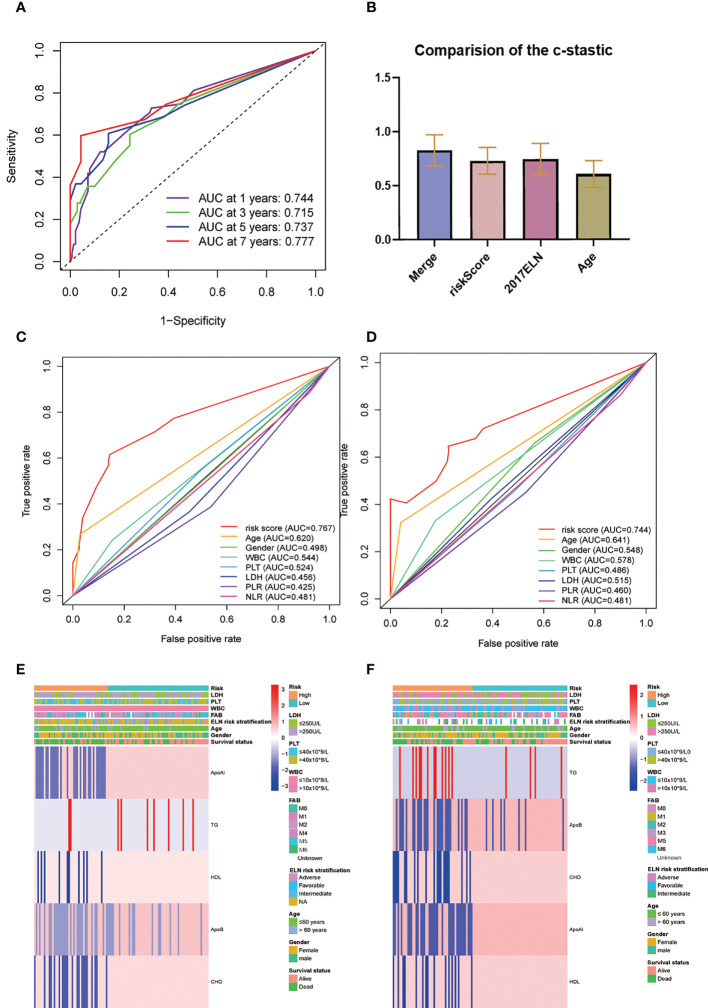
**(A)** Time-dependent ROC analysis for one-, three-, five-, and seven-year OS for the lipid prognostic model. **(B)** C-indices were compared by combining the lipid prognostic model with the 2017 ELN cytogenetic risk classification and others. A higher C-index indicates greater precision in prediction. **(C, D)** AUCs of the five-year risk score model in the training **(C)** and validation **(D)** cohorts were significantly different from other clinical indicators. **(E, F)** Heatmap of the five-lipid prognostic factor and other clinicopathological characteristics in different risk scores for the training **(E)** and the validation **(F)** cohorts.

The AUC of the RS model for the whole group for five-year OS was 0.737 and 0.723 for 10-year OS (**Figure 5A**). The consistency indices (C indices) were assessed for the prognostic co-variates and RSs alone and in combination. A higher C-index represents more accurate assessment results. It was found that the nomogram performed the best **(** **Figure 5B**). When compared with other clinical indicators, the RS had the best predictive accuracy: the AUCs of the RS for five-year OS were 0.767 and 0.744 in the TC and VC, respectively, while those for age were 0.620 and 0.641, and for WBC were 0.544 and 0.578. (**Figures 5C, D**). Overall presentation of the distribution the five-lipid prognostic factor and other clinicopathological characteristics in different risk scores for the TC and the VC were displayed by the heatmap (**Figures 5E, F**).

**Figure 6:**
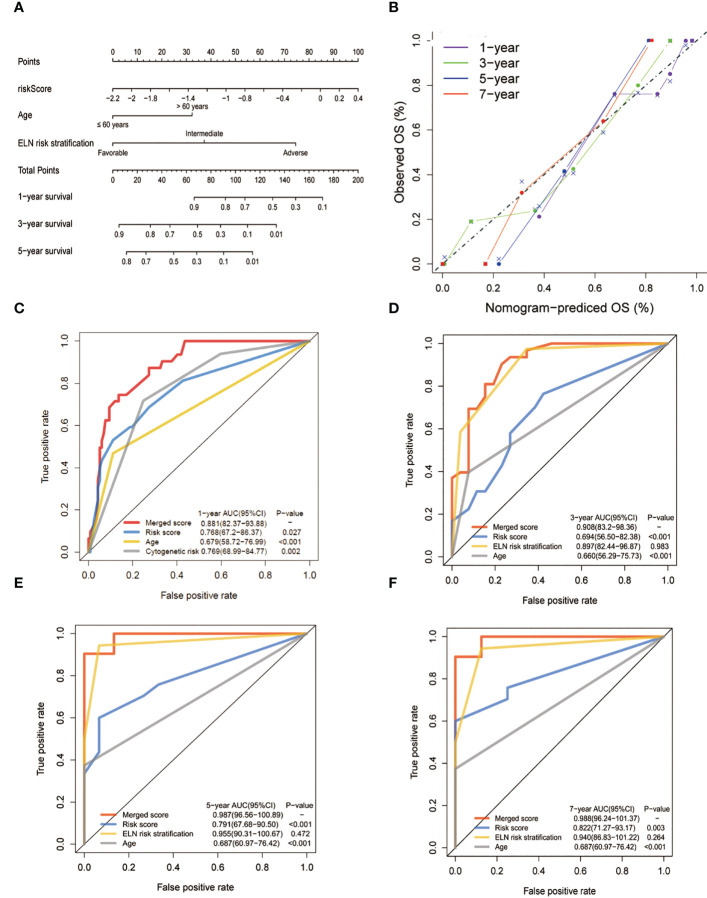
Construction and validation of the nomogram for prediction of OS in patients with AML. **(A)** The nomogram plot was constructed based on the lipid risk score and the 2017 ELN cytogenetic risk classification. **(B)** Calibration plot of the nomogram. **(C-F)** AUCs of one-,three-, five-, and seven-year OS. The lipid profile risk score showed greater predictive accuracy than the 2017 ELN classification for one-year OS while the merged model showed greater predictive accuracy for all observed years.

To create a better means of evaluation, the cytogenetic results, age, and the metabolic model were integrated into a nomogram (**Figure 6A**). The calibration plots exhibited satisfactory nomogram performance in estimating the one-, three-, five-, and seven-year OS (**Figure 6B**). The AUCs of the overall scores for the one-, three-, five-, and seven-year OS were 0.881, 0.908, 0.987, and 0.988, respectively, which were significantly higher than the 2017 ELN cytogenetic classification or the age alone, indicating that the nomogram was more effective in predicting OS than traditional prognostic markers (**Figures 6C–F**).

## Discussion

In this study, we focused on the relationship between cellular dyslipidemia and clinical outcome in AML patients. A model for predicting survival based on retrospective biochemical and clinical data was constructed and confirmed. The model provided an RS calculated from four lipid indices (Apo A-I, Apo B, CHO, TG, and HDL) which are routinely measured in blood biochemical tests. After patients were stratified into HR and LR groups according to the lipid results, it was found that the HR group had poor prognoses. Due to the physiological characteristics of disease-sustaining leukemic stem cells, we further combined the RS with other factors including the broadly applied 2017 ELN cytogenetic classification to generate a nomogram. The time-dependent ROC curve illustrated that the merged model was more effective and that the merge significantly enhanced the predictive accuracy.

Several studies have reported abnormalities in the plasma lipid profiles of leukemia patients (8, 9); however, none of these has systematically demonstrated a link between the lipid profile and prognosis. In this study, we sought to use a layer-by-layer analysis to elucidate the association between lipid profiles and OS of leukemia patients, finding a specific connection. To reduce the influence of variations in treatment, we included only patients who had been treated with the standard induction regimen for AML. We created a new risk classification model based on clinical metabolic data and combined it with other prognostic factors for clinical application. Further validation demonstrated that the RS showed significant prognostic differentiation.

After many years of investigation, the diagnosis and treatment of leukemia have matured and been systematized, with hematopoietic stem cell transplantation significantly extending the survival of patients. However, leukemia still has an extremely high mortality rate (1). More comprehensive and precise risk stratification and treatment strategies are urgently needed. The unchecked proliferation of malignant tumor cells creates disordered metabolism in the cells, and recent *in vitro* studies have suggested that certain genetic changes in leukemia cells are associated with enhanced dynamics and metabolism of lipid species in AML (11). The pathogenesis and chemoresistance of leukemia are closely related to abnormal tumor cell metabolic microenvironments including disorders in the lipid profile reflected by serum lipid levels, which has both prognostic and therapeutic target value.

Our study analyzed the relationship between cholesterol, triglycerides, apolipoproteins, and other indicators of body lipid metabolism, and clinical survival outcomes in acute myeloid leukemia from the perspective of macroscopic laboratory indicators. The formula for calculating the risk score derived from the LASSO analysis showed that the triglyceride (TG) level had a greater impact on the assigned score. Triglycerides play a pivotal role in the synthesis and utilization of fatty acids, which play important roles in the use of cellular energy, and an intermediate product of TG synthesis and utilization, diacylglycerol, acts as a second messenger in cellular signaling (12). It has been verified that AML cancer cells are dependent on very long-chain fatty acids for their energy supply (13), which may be related to the abnormal micro-metabolic environment of tumor cells. Increased serum TG levels are frequently observed in leukemia patients, including pediatric patients (14). ApoA-1 is the main component of HDL, which is critical for lipid metabolism and inflammation. Apo B is also synthesized by the liver and is the main structural protein of LDL-CHOL, accounting for about 97% of the total protein content of LDL-CHOL (15). It has been proposed that the products derived from lipoprotein-peroxide interactions could contribute to mutagenicity and carcinogenicity in cells (16). In the present study, we found that the serum Apo A-1 and Apo B levels were positively associated with OS in AML patients. Significant reductions in serum LDL levels have also been reported in AML patients (17). The results of these studies are generally in good agreement with the findings of our analysis.

Several studies have suggested that leukemia stem cells (LSCs) with the potential for self-renewal are responsible for disease sustainability, and traditional cyto-cycling or cytotoxic drugs are unable to target these relapse-related stem cells (18). It has been shown that after administration of drugs that disrupt cellular lipid homeostasis, it is possible to specifically kill LSCs without affecting normal hematopoietic stem cells (19). This may be related to the observation that LCSs from patients with relapsed AML are able to undergo oxidative phosphorylation for energy supply through fatty acid metabolism, whereas this process only occurs through amino acid metabolism in LSCs from novo AML patients (20). These discoveries suggest new directions for precision-targeting of the leukemic metabolic microenvironment, while several studies have reported re-normalization of serum lipid level after standard chemotherapy (21). At the same time, several investigations into the targeting of lipid metabolism at the molecular level and the identification of novel therapeutic targets in AML cells have confirmed a specific association between abnormal metabolism, such as lipid peroxidation, and the biological behavior of tumor cells. The form of cell death associated with lipid peroxidation has also been linked to ferroptosis in tumor cells (22). These results have led us to speculate about the role that targets related to metabolic processes such as fatty acid energy supply and lipid peroxidation could play in the future treatment of AML.

On the other hand, since most of the chemotherapy drugs used for treating leukemia are highly toxic, their effects on liver and kidney functions cannot be ignored (23). The standard induction chemotherapy regimen for AML patients is based on high doses of anthracyclines and cytarabine. Whether the effects of abnormal lipid levels before treatment combined with the application of cytotoxic chemotherapeutic agents on the metabolic function of the body influences patient survival outcomes require further investigation. Our analysis of the data failed to elucidate clear differences in serum lipid markers before and after treatment due to some missing data. Research to further clarify the relationships and mechanisms between them is required.

As described above, although the significance of lipid parameters in predicting survival has been confirmed, there were, nevertheless, some limitations to this process. When time-dependent ROC curves were applied in the entire patient cohort, we noticed that the AUCs of the three- and five-year RS values were below those of the 2017 ELN risk classification (0.761 vs. 0.903 and 0.763 vs. 0.909, respectively). ELN staging incorporates a variety of AML prognosis-related genes, including gene mutations related to lipid metabolism, and has been demonstrated to be a complete acute myeloid prognosis-stratified management system (24, 25). while the intention of our RS is to illustrate the relationship between serum lipid levels and prognosis in AML patients from a macroscopic blood biochemistry perspective. In general, this novel RS model supports the 2017 ELN cytogenetic risk classification through integration into a nomogram. Research conducted in Japan has reported that statins used for the control of blood lipid levels reduced the transcription of AML-1A, a MIP-1α transcription factor (26), suggesting there are many associations between serum lipid levels and AML prognosis that are worth exploring. Recent advances in high-throughput sequencing (next-generation sequencing) technology have made it possible to detect precise mutations associated with AML, not only for the highly sensitive detection of molecular measurable residual disease (MRD) after chemotherapy but also for the detection of mutations at loci that can determine the prognosis of the disease, such as FLT3 and EVI1 (27). After further research to elucidate the mechanisms linking cellular oxidative energy supply processes such as lipid metabolism to the biological behaviors of AML tumor cells, perhaps next-generation sequencing could also be applied to the detection of metabolism-related gene mutations, including lipids, to guide precision therapy and early clinical intervention in AML patients.

Despite the interesting data, this research has certain limitations. First, the data were collected over an extended period and it was not possible to evaluate them systematically using a unified assessment program. Second, all patient data were from a single institution, which may introduce potential bias. The sample population was relatively small and additional studies are warranted to assess whether the optimal threshold is applicable on a wider scale. Additional large, prospective, multicenter investigations would be needed to confirm our conclusions, and a quantifiable, clinically guided, and simply operationalized risk scoring system is currently lacking. Lastly, the possible molecular biological mechanisms involving lipid metabolism in AML development and progression and its therapeutic value remain to be systematically explored in a more specific manner.

## Conclusion

Using real-world clinical data as a foundation, a model was constructed using data on serum lipid profiles to estimate OS in AML patients. Lipid profiles can thus be used as new prognostic indicators to enhance the predictive precision of traditional factors including the revised 2017 ELN genetic risk stratification of AML and may promote the future study of incorporating lipid metabolism in the precision regulation of treatment regimens for patients with AML.

## Data availability statement

The original contributions presented in the study are included in the article/**Supplementary Material**. Further inquiries can be directed to the corresponding authors.

## Ethics statement

The studies involving human participants were reviewed and approved by Ethics Committees of Sun Yat-sen University Cancer Center. Written informed consent to participate in this study was provided by the participants’ legal guardian/next of kin. Written informed consent was obtained from the individual(s) for the publication of any potentially identifiable images or data included in this article.

## Author contributions

Study concept and design: HZW, SB, and RS; data collecting: JW and SL; statistical analysis: HZW, RS, and SB; figure and tables preparation: SB, HZW, and RS; writing-original draft: SB and HZW; data and tables inspection and validation: SB and BF; project administration: YL and HW; work supervision: YL and HW; Writing-review and editing: YL and HW; Funding acquisition; YL. Critical revision of the manuscript for important intellectual content: all authors. All authors contributed to the article and approved the submitted version.

## Supplementary material

Supplementary Figure 1 Construction of the lipid profile-related model. A. 1000 bootstrap replicates by LASSO regression analysis for variable selection. B. LASSO coefficients of lipids and apolipoproteins. Each curve represents a factor.
