# Supplementary figures and images for: Lipid profile as a novel prognostic predictor for patients with acute myeloid leukemia
Source: Front Oncol. 2023 Jan 31;13:950732. doi: 10.3389/fonc.2023.950732 (PMC9927215; doi:10.3389/fonc.2023.950732)

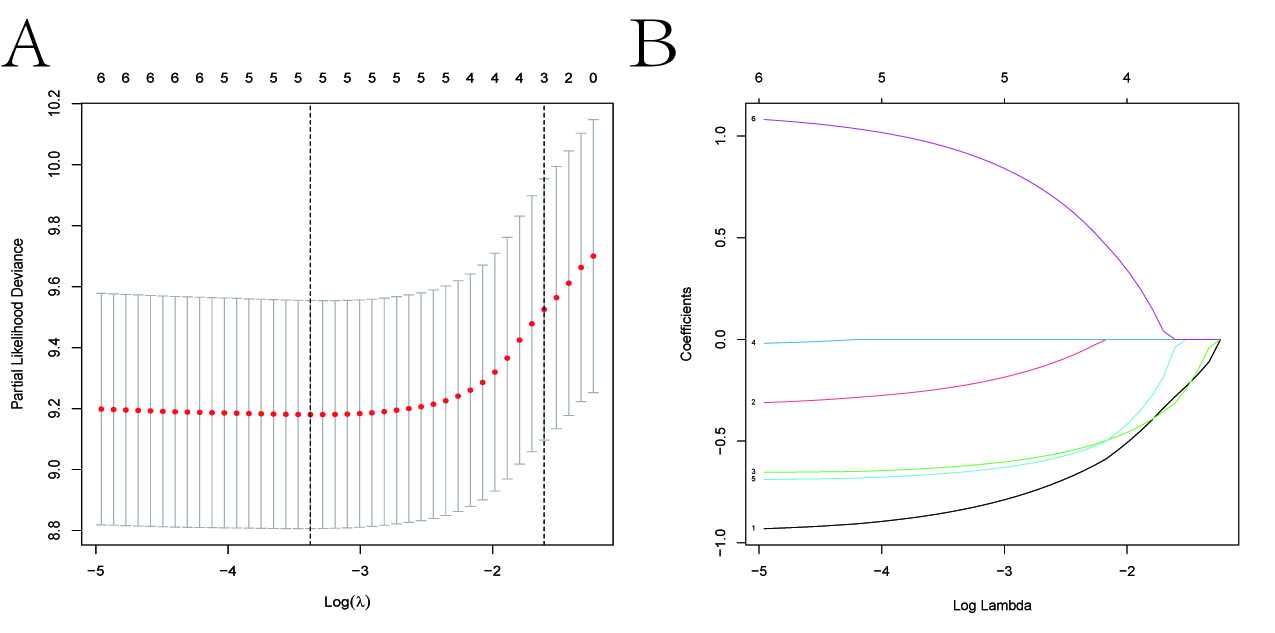

Supplement: Supplementary Figure 1 — Construction of the lipid profile-related model. A. 1000 bootstrap replicates by LASSO regression analysis for variable selection. B. LASSO coefficients of lipids and apolipoproteins. Each curve represents a factor. [file Image_1.tif]
