# Supplementary material for: Lipid profile as a novel prognostic predictor for patients with acute myeloid leukemia
Source: Front Oncol. 2023 Jan 31;13:950732. doi: 10.3389/fonc.2023.950732 (PMC9927215; doi:10.3389/fonc.2023.950732)
Supplement: Supplementary file 2 [file Table_1.docx]

| **Supplemental Table 1. The baseline characteristics and clinicopathological features of patients with AML.** |
| --- |
| **Variables No. of patients (%)** median(mean ± sd )  **N=214** |

**age**

≤60 175(81.8) 45±16

>60 39(18.2)

**gender**

male 91(42.5)

female 123(57.5)

**WBC**

≤76 189(81.8) 39.1±54

>76 42(18.2)

**Apo AI**

≤0.7 72(33.6) 0.94±0.31

>0.7 142(66.4)

**Apo B**

≤0.65 90(29.9) 0.74±0.22

＞0.65 124(60.1)

**CHO**

≤2.67 52(24.3) 3.54±1.02

>2.67 162(75.7)

**TG**

≤2.55 178(83.2) 1.64±0.99

>2.55 36(16.8)

**HDL**

≤0.45 40(18.7) 0.81±0.39

>0.45 174(81.3)

**LDL**

≤1.43 39(18.2) 2.81±9.76

>1.43 175(81.8)

**LDH**

<187.9 127(59.3) 509.83±636.48

≥187.9 87(40.7)

**BMI**

< 19.3 59(27.6) 21.5±3.2

≥19.3 155(72.4)

**2017 ELN Cytogenetic**

**risk classification**

Favorable 48（22.4） NA

Intermediate 49（22.9） NA

Adverse 63（29.4）

Unable to access 54（25.2） NA

|  |
| --- |
| **Variables No. of patients (%)** median(mean ± sd )  **N=231** |

**Initial therapy** NA

Regimens containing

anthracyclines and cytarabine (DA/IDA) 214(92.6)

Other regimens including 9(3.9)

decitabine, etoposide or hydroxyurea

Without treatment or unable to access 8(3.5)

Abbreviations: LDH lactate dehydrogenase; TC total cholesterol; TG tri-glyceride; LDL low-density lipoprotein cholesterol; HDL high-density lipoprotein cholesterol; Apo B Apolipoprotein B; Apo A-I Apo Apolipoprotein A-I.
